# Development of a robust and convenient dual-reporter high-throughput screening assay for SARS-CoV-2 antiviral drug discovery

**DOI:** 10.1016/j.antiviral.2022.105506

**Published:** 2023-02

**Authors:** Winston Chiu, Joost Schepers, Thibault Francken, Laura Vangeel, Kayvan Abbasi, Dirk Jochmans, Steven De Jonghe, Hendrik Jan Thibaut, Volker Thiel, Johan Neyts, Manon Laporte, Pieter Leyssen

**Affiliations:** a KU Leuven, Department of Microbiology, Immunology and Transplantation, Rega Institute, Laboratory of Virology and Chemotherapy, Herestraat 49 – box 1043, 3000, Leuven, Belgium; b KU Leuven Department of Microbiology, Immunology and Transplantation, Rega Institute, Laboratory of Virology and Chemotherapy, Translational Platform Virology and Chemotherapy, Gaston Geenslaan 2, 3001, Leuven, Belgium; c Institute of Virology and Immunology (IVI), Bern, Switzerland; d Department of Infectious Diseases and Pathobiology, Vetsuisse Faculty, University of Bern, Bern, Switzerland

**Keywords:** High-throughput screening, Reporter SARS-CoV-2, Reporter A549, Assay development

## Abstract

Massive efforts on both vaccine development and antiviral research were launched to combat the new severe acute respiratory syndrome coronavirus 2 (SARS-CoV-2). We contributed, amongst others, by the development of a high-throughput screening (HTS) antiviral assay against SARS-CoV-2 using a fully automated, high-containment robot system. Here, we describe the development of this novel, convenient and phenotypic dual-reporter virus-cell-based high-content imaging assay using the A549+hACE2+TMPRSS2_mCherry reporter lung carcinoma cell line and an ancestral SARS-CoV-2_Wuhan_mNeonGreen reporter virus. Briefly, by means of clonal selection, a host cell subclone was selected that (i) efficiently supports replication of the reporter virus with high expression, upon infection, of the NeonGreen fluorescent reporter protein, (ii) that is not affected by virus-induced cytopathogenic effects and, (iii) that expresses a strong fluorescent mCherry signal in the nucleus. The selected clone matched these criteria with an infection rate on average of 75% with limited cell death. The average (R)Z′-factors of the assay plates were all >0.8, which indicates a robust assay suitable for HTS purposes. A selection of reference compounds that inhibits SARS-CoV-2 replication *in vitro* were used to validate this novel dual-reporter assay and confirms the data reported in the literature. This assay is a convenient and powerful tool for HTS of large compound libraries against SARS-CoV-2.

## 1 Introduction

Globally, the ongoing COVID-19 pandemic, caused by severe acute respiratory syndrome coronavirus 2 (SARS-CoV-2) still results in approximately 3 million cases and 10,000 deaths per day (https://covid19.who.int/) (October 2022) (“WHO Coronavirus, 2022). The clinical spectrum varies from asymptomatic infections to severe pulmonary disease, possibly resulting in death or long-term consequences, such as long-covid (“ZOE COVID Study, 2022), (Zaim et al., 2020). Effective vaccines were developed and administered in record times. However, immunity by vaccination wanes in part because of the emergence of new variants, leading to a need for boosters and updated vaccines (Fiolet et al., 2022).

Recently, the first oral antivirals for the treatment of SARS-CoV-2 infections, molnupiravir and nirmatrelvir–ritonavir, have been approved (Agostini et al., 2019; Sheahan et al., 2020; Owen et al., 2021). Their antiviral activity is independent of the variant of concern (Vangeel et al., 2022). Molnupiravir has rather low efficacy in the clinical setting and nirmatrelvir needs to be combined with ritonavir, which limits its use, because of drug-drug interactions in multi-medicated patients (“Paxlovid Drug-Drug Interactions, 2022), (Jayk Bernal et al., 2022). In addition, the potential development of resistance is a concern (Jochmans et al., 2022), (Iketani et al., 2022). There is hence still an urgent need to develop novel therapeutic agents with potent, selective antiviral activity, with another mechanism of action than the currently available drugs against SARS-CoV-2 that can be used to treat or prevent COVID-19.

A valid and efficient method to identify new classes of molecules is by high-throughput screening (HTS) of large small-molecule libraries in a large-scale, cost-effective way. At the start of the COVID-19 outbreak and to find a therapy that could save patients' lives, several HTS assays were developed to screen drug repurposing compound libraries for antiviral activity against SARS-CoV-2, including collections of approved and investigational drugs with known toxicity and pharmacokinetic profiles (Riva et al., 2020; Bakowski et al., 2021; Chiu et al., 2022; Zaliani et al., 2022).

A widely used phenotypic HTS strategy is cell-based imaging. This requires typically long and tedious staining protocols with either fluorescent-labeled antibodies that target a specific viral protein or with fluorescent dyes that selectively stain intracellular structures. The generation of cell lines that are engineered to express a fluorescent reporter protein drastically improved the throughput of phenotypic HTS assays, since the cellular factor staining steps can be omitted (Chalfie et al., 1994), (Kanda et al., 1998). In addition, utilizing reporter viruses that are engineered to express a fluorescent marker protein upon infection eliminates the need for any further staining steps (Zou et al., 2011; Rimmelzwaan et al., 2011; Shang et al., 2013; Chiem et al., 2021; Ter Horst et al., 2021). Therefore, combining reporter cells with reporter viruses offers a convenient and robust strategy for phenotypic HTS.

HTS campaigns that involve infectious SARS-CoV-2 virus can only be performed in biosafety level 3 (BSL-3) containment (CDC, 2020). Although various HTS campaigns on SARS-CoV-2 have been performed over the past years, it is not straightforward or convenient to perform this in a BSL-3 environment (Riva et al., 2020), (Zaliani et al., 2022). Specialized facilities, biosafety protocols and regulatory requirements limit work with these live pathogens (“Biosafety in Microbiological and Biomedical, 2021). To improve efficiency and throughput, many labs used automation instruments such as non-contact liquid handlers, plate washers, and different read-out instruments (Chiu et al., 2022), (White et al., 2016), (Chung et al., 2010). However, to our knowledge, none of the BSL-3 laboratories around the world have a fully automated BSL-3 isolator system that has been specifically designed for HTS and high-throughput research on and with live pathogens of higher biosafety level (Caps-It, 2022).

The VeroE6 cell line has been the most prominent cell line used in the early phase of the pandemic for HTS campaigns against SARS-CoV-2. This cell line shows extensive cytopathic effects (CPE) upon infection with SARS-CoV-2 and hence is suitable for the establishment of an antiviral assay (Chiu et al., 2022), (Zaliani et al., 2022), (Ivens et al., 2005), (Saul et al., 2021). However, being a monkey kidney-derived cell line, its relevance is debated since the major target of SARS-CoV-2 are respiratory epithelial cells. Furthermore, VeroE6 cells typically express high levels of the efflux transporter multi-drug resistance 1 (MDR1) or P-glycoprotein (P-gp). As a consequence, antiviral assays are typically performed in presence of an efflux inhibitor to avoid misinterpretation of the data (Zhu et al., 2022). A final drawback of the VeroE6 cell line is that SARS-CoV-2 acquires several genetic changes when propagated in these cells, especially in the Spike protein (Liu et al., 2020).

A human airway epithelial cell line, such as the A549 cell line, overexpressing the SARS-CoV-2 angtiotensin converting enzyme 2 (ACE2) receptor and the spike priming protease transmembrane serine protease 2 (TMPRSS2) offers an alternative and physiologically more relevant model when compared to the VeroE6 cell line (Li et al., 2003), (Hoffmann et al., 2020). The A549 cell line is highly susceptible to SARS-CoV-2 infection and can be used for HTS.

We here report on the development and optimization of an unique dual-reporter antiviral assay using the A549_+hACE2+hTMPRSS2__mCherry reporter cell line and a SARS-CoV-2_mNeonGreen reporter virus as an efficient, convenient, robust test system, which is suitable for *in vitro* HTS for antiviral drug discovery against SARS-CoV-2. To validate this assay, we used several reference compounds with known antiviral activity against SARS-CoV-2 replication *in vitro*.

## 2 Materials and methods

### 2.1 Cell lines

Human lung carcinoma cells, A549, overexpressing human ACE2 and human TMPRSS2 receptors were purchased from Invivogen (Cat no.: a549-hace2tpsa). A549^+hACE2+hTMPRSS2^_mCherry cells were cultured in Dulbecco's modified Eagle's medium (DMEM, Thermo Fisher Scientific) supplemented with 10% v/v heat-inactivated fetal bovine serum (FBS HI, Biowest), 300 μg/mL hygromycin (Invivogen), 0.5 μg/mL puromycin (Invivogen) and 10 μg/mL blasticidin (Invivogen) at 37 °C and 5% CO_2_. Antiviral assays with A549^+hACE2+hTMPRSS2^_mCherry cells were performed in Mucilair medium (Epithelix).

### 2.2 Viruses

Recombinant SARS-CoV-2_mNeonGreen virus (Wuhan strain) was a kind gift from Prof. Dr. Volker Thiel (University of Bern, Switzerland) (Thi Nhu Thao et al., 2020). Virus stocks were generated by passaging the virus on Calu-3 cells followed by production of a screening virus stock on A549^+hACE2+hTMPRSS2^ cells. Full genome sequencing was performed using MinION (Oxford Nanopore Technologies).

The infectious titer was determined by microscopic imaging of mNeonGreen fluorescent protein in infected cells and calculated using the Spearman-Kärber method. Briefly, 384-well plates (Cellstar® μClear, Greiner Bio-One, Cat: 781090) were seeded with 4.000 cells/well and incubated overnight. A 5-fold serial dilution of SARS-CoV-2_mNeonGreen virus, starting at a dilution of 1/10, was added to the plate and incubated for 3 additional days after which a high content imaging read-out was performed on the plate.

Virus infection experiments and high content imaging read-out was conducted in the Caps-It system research infrastructure as installed in the biosafety level 3 facilities of the Rega Institute (KU Leuven, Belgium; License number: AMV 30112018 SBB 219 2018 0892 and AMV 23102017 SBB 219 2017 0589).

### 2.3 Generation of a stable A549^+hACE2+hTMPRSS2^ mCherry cell line

A549^+hACE2+hTMPRSS2^ were transduced using lentiviral vectors containing a pLenti-H2B-mCherry-P2A-BlastR cassette allowing stable expression of fluorescent mCherry in the nuclei. The pLenti‐H2B-mCherry‐2A‐Blasticidin was modified from the pLenti-Cas9-2A-Blasticidin vector (Addgene 73310) by replacing the Cas9 expression sequence with the coding sequence of H2B-mCherry (Integrated DNA Technologies) using the NEBuilder HiFi DNA Assembly kit. The resulting vector was used to make lentiviral particles, as described previously (Baggen et al., 2021). Briefly, HEK293T cells were transfected using X-TremeGENE 9 (Roche) with the pLenti‐H2B-mCherry‐2A‐Blasticidin and the lentiviral packaging plasmids pMD2.G and psPAX2 to generate lentiviral particles coated with the vesicular stomatitis virus G (VSV-G) protein. After 24 h incubation, the medium was replaced by DMEM supplemented with 1.1% bovine serum albumin. The supernatant containing lentiviral particles was harvested 72 h after transfection and stored at −80 °C. A549 cells were transduced with the lentiviral stock in the presence of polybrene (8 μg/mL). After 24 h, medium was removed and replaced by medium containing blasticidin (10 μg/mL) and incubated for an additional 48 h.

The polyclonal cell culture was clonally purified using a limiting dilution strategy. In short, cells were seeded in 96-well plates at a density of 0.5 cell/well. The cells were monitored daily on FLoid™ Cell Imaging Station (Thermo Fisher Scientific) for mCherry signal. DMEM containing 10% FBS and 10 μg/mL blasticidin was refreshed twice a week. Cells with low mCherry signal, impaired cell growth or aberrant morphology were discarded. Upon reaching 50%–70% confluency in the well, cells were trypsinized and transferred to a 24-well plate and after expansion to a 6-well plate to be finally seeded in a T-25 flask. Each clone was assessed on morphology, growth kinetics, and fluorescent intensity. Ultimately, eight clones were selected for viral infectivity studies.

### 2.4 Viral infectivity studies

Eight A549^+hACE2+hTMPRSS2^_mCherry clones were subjected to viral infectivity assays using the recombinant SARS-CoV-2_mNeonGreen. For this, cells were seeded in 96-well plates one day before infection. Subsequently, cells were infected with a 5-fold virus dilution series (ranging from 10^−1^ to 10^−7^). The infected cultures were incubated at 37 °C and 5% CO_2_ for six days and microscopic images were collected every 24 h to assess the (possible) development of CPE and to monitor the appearance and intensity of the mNeonGreen signal as indicators of viral replication.

Additional parameters including cell seeding number, virus input, read-out day, assay medium, and pre-infection culture days were varied and evaluated. Intra- and interplate variability as well as Z′-factor were calculated. Z′ is a statistical parameter that measures assay quality and shows the signal range between the positive controls and negative controls. The equation for Z′ is shown below where σ is the standard deviation and μ is the mean of the positive controls (p) and negative controls (n). A Z′ of >0.5 is considered a qualitative good assay (Zhang et al., 1999).

$$Z^{\prime }=1-\frac{3\sigma_{p}+3\sigma_{n}}{\left|\mu_{p}-\mu_{n}\right|}$$

Technical replicates were normalized and averaged for each experiment. The assays with the most promising cell clones were further optimized in 384-well plate format and the same quality parameters as mentioned above were assessed. Graphpad Prism version 9.3.1 and Genedata Screener version 19.0.0 Standalone were used for data analysis.

### 2.5 Automation and high-content imaging

Image acquisition and analyses were performed using the Arrayscan XTI (Thermo Fisher Scientific) and processes were automated using the Caps-It research infrastructure (Rega Institute, KU Leuven, Belgium). The Caps-It is a fully automated robotic system enclosed in an isolator which facilitates HTS on high biosafety organisms (Caps-It, 2022).

**Fig. 1:**
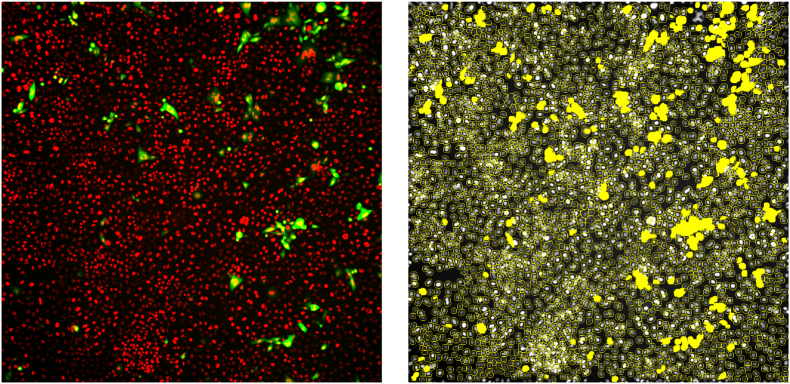
A high-content imaging read-out. Cells are encircled in yellow lines and counted as objects while virus replication represented by mNeonGreen signal is detected as yellow spots. A yellow spot within an object will be counted as one infected cell by the image analysis protocol.

An automated workflow was designed starting from virus infection until decontamination. Briefly, plates already containing the appropriate cell number in medium were imported into the Caps-It system after which the virus was added using an automated contactless liquid handling protocol (EVO100, Tecan). After infection, the plates were transported to the incubators before high-content imaging read-out (Fig. 1) was performed on set time points. Automatic image acquisition protocols were implemented for multiple measurements spread over different time points of the assay plates.

For the A549^+hACE2+hTMPRSS2^ dual reporter assay, a 5× objective was used with 2 imaging channels set on 560-23_BGRFRN_BGRFRN and 485-25_BGRFRN_BGRFRN. An in-house algorithm was created for cell counting, segmentation, and validation for calculation of the percentage infected cells. A wide array of output features was exported but only two features (cell count and percentage of virus-infected cells) were used for data analysis.

### 2.6 384-well reference compound validation screening

For validation of the A549^+hACE2+hTMPRSS2^ dual reporter assay, two reference compounds with known antiviral activity against SARS-CoV-2 were used: GS-441524 (an inhibitor of the RNA-dependent RNA polymerase and the parent nucleoside of remdesivir), and PF-00835231 (an inhibitor of the 3C-like cysteine protease (3 CLpro) also known as Main protease (Mpro) and the lead molecule of nirmatrelvir). Cells were seeded in 384-well plates at 4.000 cells/well and compounds dissolved in DMSO were added in a 10-fold serial dilution of 1/3 starting from a final concentration of 10 μM. After overnight incubation, virus was added to the wells (MOI = 0.002). Plates were incubated for three and four days after which a high-content imaging read-out was performed. In addition, the half-maximal effective concentration (EC_50_) was calculated for each compound and compared with data reported in the literature.

## 3 Results

### 3.1 Generation of A549^+hACE2+hTMPRSS2^_mCherry cell line

**Fig. 2:**
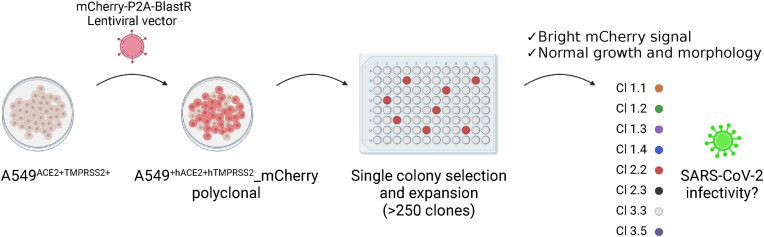
Schematic overview of the lentiviral transduction and clonal selection of A549^+hACE2+hTMPRSS2^_mCherry cells. This figure was created with BioRender.com.

The A549^+hACE2+hTMPRSS2^_mCherry cell line was engineered using lentiviral transduction and a clonal selection procedure was performed over several 96-well plates. Over 250 clones were generated and finally, eight clones were retained based on morphology, the intensity of the fluorescent signal, and growth kinetics (Fig. 2). Cells showing aberrant morphology compared to the non-transduced cells were discarded. In addition, cells growing in individual clusters instead of forming monolayers were also removed from the clonal selection procedure. Cells showing no or too faint fluorescent signal were discarded, as well as cells showing an inconsistent fluorescent signal throughout the cell population. The growth kinetics were determined for each clonal population. Cells showing an irregular proliferation rate were discarded. The final eight clones showed morphological characteristics similar to standard A549 cells and exhibited a strong fluorescent mCherry signal requiring minimal light exposure during imaging.

### 3.2 Viral infectivity assessment of generated clones

**Fig. 3:**
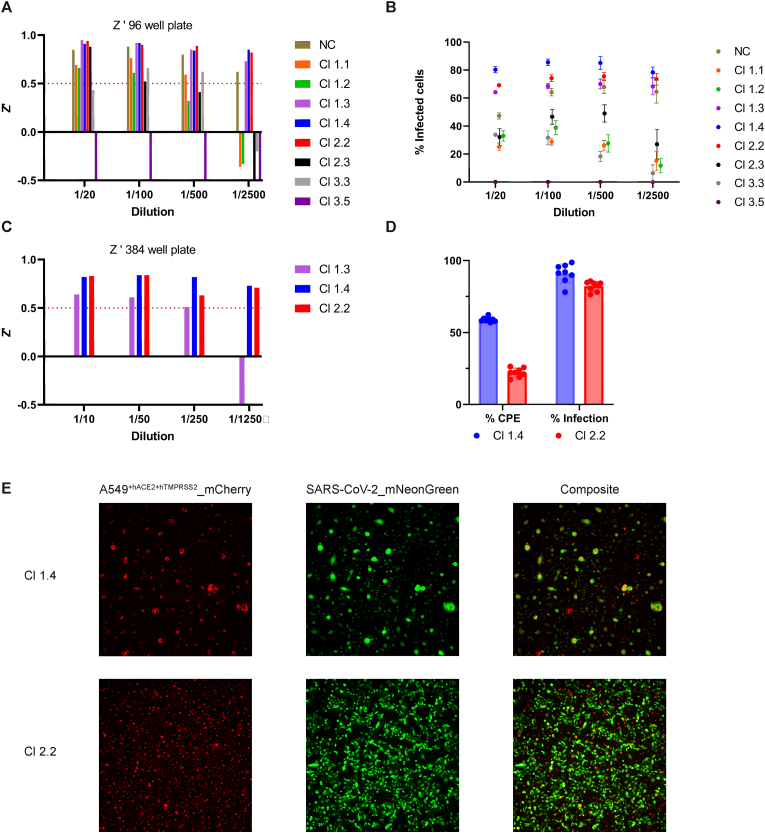
A. Z′ of the eight selected clones calculated from uninfected cell cells and cells infected with SARS-CoV-2_mNeonGreen on day three post infection in 96-well format. The non-cloned cells (NC) and clones (Cl) 1.3, 1.4 and 2.2 showed a Z’ > 0.5 at 1/2500 final dilution. Cells were seeded at 15.000 cells/well and infected with different virus dilutions. Eight technical replicates were performed for each dilution. B. Infection rate of SARS-CoV-2_mNeonGreen on the eight clones at different virus dilutions. A high-content imaging read-out was performed on day three post infection to count the cells containing mNeonGreen, which were indicated as ‘%infected cells’. Clones (Cl) 1.3, 1.4 and 2.2 showed consistent %infected cells of >60% at all dilutions and were selected for further optimization. Clone 3.5 did not permit infection. The graph shows 8 technical replicates and error bars represent the standard deviation. C. Z′ of the three remaining clones calculated from uninfected cell cells and cells infected with SARS-CoV-2_mNeonGreen on day three post infection in 384-well format. Cells were seeded at 4.000 cells/well and infected with different virus dilutions (n = 32). Cl 1.4 and Cl 2.2 showed Z’ > 0.5 in all virus dilutions. D. Cytopathogenic effect (CPE) and % infection comparison between Cl 1.4 and Cl 2.2. Cells were infected with SARS-CoV-2_mNeonGreen at MOI 0.002 (n = 8). Both clones show similar % infection. Cl 1.4 show significantly more % CPE than Cl 2.2. Data visualized as mean ± standard deviation. E. Microscopic images from Cl 1.4 and Cl 2.2 infected with SARS-CoV-2_mNeonGreen. Left panel, the A549^+hACE2+hTMPRSS2^_mCherry cells. Cl 1.4 show virus induced CPE as big lumps and reduced cell count, while Cl 2.2 show an unimpaired monolayer. Middle panel, mNeonGreen fluorophore expressed by the cells after infection. Right panel: Composite image of both panels.

The eight generated A549^+hACE2+hTMPRSS2^_mCherry clones were subjected to viral infection with recombinant SARS-CoV-2_mNeonGreen. Cells were infected with a 5-fold serial dilution starting from a 1/20 dilution of the original virus stock to determine the infectivity efficiency. High-content imaging read-out was performed every 12 h over a period of 6 days to evaluate cell growth and morphology, viral replication, and induction of CPE in these cell clones (Cl). Seven Cls showed efficient viral replication of SARS-CoV-2_mNeonGreen. Interestingly, Cl 3.5 did not permit viral infection and/or replication. Cl 1.1, 1.2, 2.3 and in Cl 3.3, never more than 60% infection rate was achieved resulting in low Z′ values as presented in Fig. 3A and B, and were withheld from further assay optimization. The remaining three clones (1.3, 1.4 and 2.2) were further optimized in 384-well format. Cl 1.3 exhibited inconsistent mNeonGreen expression resulting in a low Z′-factor (Fig. 3C) and was therefore discarded. Cl 1.4 and 2.2 resulted in consistent >75% infection at both three and four days post-infection while retaining a Z’ > 0.5 on different virus dilutions. Next, additional parameters i.e., cell density, pre-incubation time, read-out time, assay medium, and MOI were assessed for these two clones. For the final optimization steps, both clones were compared at a final virus dilution of 1/1.000 (MOI = 0.002). Cl 1.4 and 2.2 resulted in a comparable % of infection. However, clone 1.4 had higher discrepancies in cell count between infected and non-infected controls and showed higher intra- and interplate variability (Fig. 3D and E). Finally, Cl 2.2 was selected for HTS because it resulted in consistent mNeonGreen expression and caused limited CPE, which facilitate readout after longer incubation times. Moreover, it had a comparable cellular morphology as the original A549^+hACE2+hTMPRS2^ cells. Cl 2.2, plated at 4.000 cells/well in a 384-well plate, infected with SARS-CoV-2_mNeonGreen at MOI = 0.002 and read-out on day three post infection was selected for assay validation with reference compounds.

### 3.3 Validation using reference compounds

To confirm that replication of SARS-CoV-2_mNeonGreen in the selected A549^+hACE2+hTMPRSS2^_mCherry Cl 2.2 could be inhibited, an antiviral assay was performed using the known SARS2-CoV-2 inhibitors GS-441524, a polymerase inhibitor and PF-00835231, an inhibitor of 3CLpro. Both GS-441524 and PF-00835231 resulted in antiviral activity against SARS-CoV-2_mNeonGreen in A549^+hACE2+hTMPRSS2^_mCherry cells with an EC_50_ of 1.66 ± 0.42 μM and 0.43 ± 0.08 μM respectively (Table 1). The CC_50_ was calculated simultaneously by measuring the number of cells as an indication of cellular toxicity. For both compounds, the CC_50_ was determined to be > 50 μM, resulting in a selectivity index (SI) of >30 for GS-441524 and > 115 for PF-00835231. These values were similar to those that have been reported in the literature (Abdelnabi et al., 2022), (Chang et al., 2022). In total, 32 × 384-well plates were used to confirm the robustness of this assay. Four separate runs were performed using different instruments on four different days and the plates were incubated in different incubators. In addition, one run was performed by two separate operators starting from independent cell and virus cultures. Each plate contained a quadruplicate of the dose-response conditions for GS-441524 and PF-00835231. The coefficient of variation (CV) on the EC_50_ was 25% for GS-441524 (n = 128) and 19% for PF-00835231 (n = 128). As control conditions, we used wells with infected cells and wells with uninfected cells. The average Z’ factor across all plates was 0.72 ± 0.08 (Supplementary Fig. 1). In addition, other known inhibitors of SARS-CoV-2 replication *in vitro* were assessed in 12 replicates (four intraplate replicates and three plate replicates) to confirm assay accuracy and is shown in Table 1.

**Table 1:** Effect of a selection of reference SARS-CoV-2 inhibitors on viral replication and on host cell viability.

Compound	N	EC_50_ (μM)	CC_50_ (μM)
		Mean ± SD	Mean ± SD
PF-00835231	128	0.43 ± 0.08	>50
GS-441524	128	1.66 ± 0.42	>50
Nirmatrelvir	12	0.12 ± 0.017	>50
Molnupiravir	12	1.26 ± 0.23	>50
BAY-2402234	12	0.005 ± 0.001	0.03 ± 0.01
Alisporivir	12	1.46 ± 0.13	23.75 ± 4.75
Remdesivir	12	0.04 ± 0.005	>50
Ensitrelvir	12	0.26 ± 0.043	>50
Camostat	12	>50	>50

Mean antiviral activity (EC_50_) and cytotoxicity (CC_50_) values of the reference compounds used for assay validation. N = replicates and SD = standard deviation.

## 4 Discussion

In immediate response to the COVID-19 pandemic, multiple anti-SARS-CoV-2 cell-based HTS campaigns have been set up and performed all over the world (Bakowski et al., 2021), (Chiu et al., 2022), (Choi et al., 2021), (Bardiot et al., 2022). Most of these assays used indirect measurements for viral infection such as virus-induced CPE as primary readout. Direct measurement of infection using immunofluorescent staining of viral proteins is often labour-intensive, time-consuming, and not ideal for HTS, especially when to be performed with a virus of higher biosafety level. Here, we describe a novel dual reporter HTS assay against SARS-CoV-2 using both an engineered A549^+hACE2+hTMPRSS2^_mCherry reporter cell line and a SARS-CoV-2_mNeonGreen reporter virus. Using a dual reporter assay, we established a convenient method to measure viral infection directly with limited effort and limited risk. This assay proves scalable and suitable for HTS using automation systems.

We evaluated in this assay the antiviral activity against SARS-CoV-2 replication of a selection of SARS-CoV-2 reference inhibitors. The results obtained were concordant to reports from other publications (Agostini et al., 2019), (Vangeel et al., 2022), (Chang et al., 2022), (Patten et al., 2022), (Softic et al., 2020). An additional benefit of our assay is that it allows simultaneous read-out of both the antiviral potential and cytotoxicity caused by the compound. Cytostatic effects could also be observed in a host-targeting antiviral compound, the dihydroorotate dehydrogenase (DHODH) inhibitor BAY-2402234, which targets the host's pyrimidine synthesis pathway (Supplemental Fig. 2) (Xiong et al., 2020).

Several teams established antiviral assays, and carried out screens in cell lines such as VeroE6, HuH7, Caco2 and Calu-3 (Chiu et al., 2022), (Dittmar et al., 2021). The A549 cell line used here are lung epithelial cells considered relevant for infection studies with respiratory pathogens. Overexpression of ACE2 and TMPRSS2 proteins on the cell membrane, which they lack, is however required for efficient viral entry and priming of SARS-CoV-2 spike. Through lentiviral transduction, we engineered a commercially available A549^+hACE2+hTMPRSS2^ cell line to express a fluorescent H2B_mCherry protein in the nucleus. The most optimal clone was selected based on fluorescent intensity, morphology, growth rate, and infectivity. Several other respiratory viruses such as severe acute respiratory syndrome (SARS-CoV-1), parainfluenza viruses (PIVs), respiratory syncytial virus (RSV), and human metapneumovirus (HMPV) will be tested to assess infectivity and replication in these cells. Therefore, the established cell line is highly versatile and adaptable for screening of antiviral compounds against an array of respiratory viruses.

Phenotypic screening assays with automated high-content imaging readout have several advantages as compared to target-based screens (e.g. protease or polymerase screening assays): (1) since the full replication cycle of the virus is covered, novel druggable targets are apt for inhibition by any of the compounds of which the antiviral potential is being assessed, (2) the effect of the compound on the host cell can be evaluated using viability readouts and cell painting assays, (3) mode of action studies can be performed by small modifications on the assay set up e.g. time-of-addition experiments.

The newly developed high-content imaging-based HTS assay in this study is automated, scalable, robust, and suitable for screening of large compound libraries. All parameters representing assay quality such as Z′, %CV, intra- and interplate variability were excellent (Zhang et al., 1999). In addition, this assay requires limited pipetting steps, significantly decreasing the biosafety risk associated with plate handling and especially the opening thereof, and image acquisition does not need any finetuning between plates. The in-house developed image analysis algorithm can process plate batches as a whole. Reproducibility is another key factor in HTS campaigns since these can take up to couple of weeks. We have tested the reproducibility of this assay by letting several lab technicians perform the entire assay independently starting from maintaining the cell culture to the final read-out. In addition, different hardware instruments such as liquid handlers and incubators were used during this ccomparative exercise. The average Z’ was 0.72 with a SD of 0.08 indicating that our assay is highly reproducible. In addition, assay robustness was taken into account during optimization by performing the read-out on day three and day four post infection. Changes in % virus infection and cell viability were monitored daily to find a read-out window of at least 24 h that ensures robust analysis since in HTS campaigns a significant time difference occurs between the first and the last processed plate.

In summary, we developed a highly efficient, robust, and convenient HTS assay for the screening of large compound libraries against SARS-CoV-2. Furthermore, the A549^+hACE2+hTMPRSS2^_mCherry cell line is suitable for studies and screens with other fluorescent-labeled respiratory viruses in an automation setting.

## Author contributions

WC: study design, experimental work, image analysis, data analysis, writing and preparing figures; JS: study design, experimental work, image analysis, data analysis and preparing figures; TF and KA: experimental work; LV: study design, writing, revision and proofreading of the manuscript; DJ: supervisory duties; SDJ: study design, revision and proofreading of the manuscript; HJT: writing and supplied the A549+hACE2+hTMPRSS2_mCherry cells; VT: supplied the SARS-CoV-2_mNeonGreen virus; JN: study design, funding, writing, revision and proofreading of the manuscript; ML: writing and preparing figures; PL: study design, supervisory duties, writing, revision and proofreading of the manuscript.

All authors read and approved final version of the manuscript.

## Declaration of competing interest

The authors declare that they have no known competing financial interests or personal relationships that could have appeared to influence the work reported in this paper.

## Data Availability

Data will be made available on request.
