# Supplementary figures and images for: Development of a robust and convenient dual-reporter high-throughput screening assay for SARS-CoV-2 antiviral drug discovery
Source: Antiviral Res. 2023 Feb;210:105506. doi: 10.1016/j.antiviral.2022.105506 (PMC9767876; doi:10.1016/j.antiviral.2022.105506)

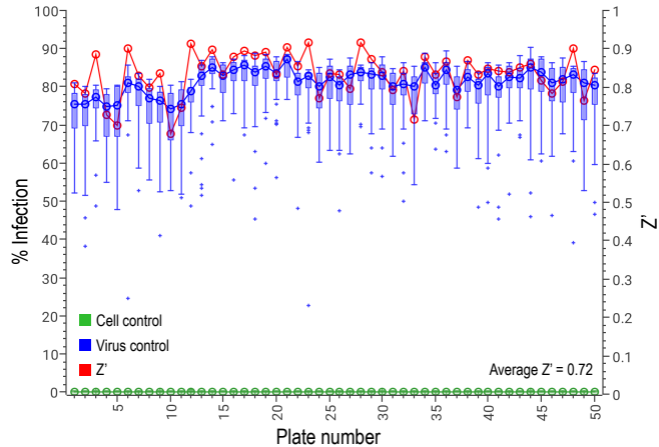

Supplement: Multimedia component 1 [file mmc1.pdf]

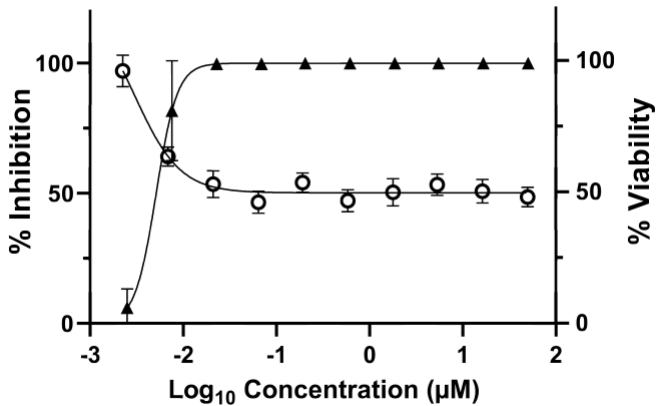

Supplement: Multimedia component 2 [file mmc2.pdf]
